# Food insecurity in older adults with subsistence jobs and its relationship with isolation conditions due to COVID-19, eating habits, and emotional symptoms, Medellin, Colombia, 2021

**DOI:** 10.47626/1679-4435-2025-1265

**Published:** 2025-09-14

**Authors:** María Osley Garzón-Duque, Fabio León Rodríguez-Ospina, Sara Isabel Sierra-Gaviria, Valeria Cano-Cano

**Affiliations:** 1 School of Medicine, Universidad CES, Medellín, Antioquia, Colombia.; 2 National School of Public Health, Universidad de Antioquia, Medellín, Antioquia, Colombia.; 3 School of Nutrition and Food Sciences, Universidad CES, Medellín, Antioquia, Colombia.

**Keywords:** food security, COVID-19, elderly, mental health, informal sector, seguridad alimentaria, COVID-19, adulto mayor, salud mental, sector informal

## Abstract

**Introduction:**

Food insecurity among older adults with subsistence jobs has been little explored in the context of the pandemic.

**Objectives:**

To identify the conditions of isolation due to COVID-19, eating habits, and emotional symptoms associated with food insecurity among older adults with subsistence jobs in Medellín, Colombia, 2021.

**Methods:**

Cross-sectional study with primary sources of information from 208 workers. The design and execution of the fieldwork were carried out in collaboration with the workers and their leaders. Results are presented for the nutritional component of workers aged ≥ 60 years who participated in the macro-project from which this product is derived.

**Results:**

68.7% were between 60 and 69 years old, and 73.0% were heads of household. Women reported lower incomes, 53.0% were without a partner, and 58.6% did not have permission to work. Men had higher consumption of cigarettes and alcohol. 97.0% isolated themselves in quarantine, 83.1% received some type of support during the pandemic, and 62.2% continued to receive it. 56% were overweight or obese, and 32.7% had severe family dysfunction. 50% saw their lives affected, and 96.6% saw their work affected by mandatory isolation. 38.0% experienced moderate/severe food insecurity, which was higher (p < 0.05) among those who did not have a partner, consumed one or two meals a day, and experienced family dysfunction. Family dysfunction and consuming one or two meals a day contributed to explaining food insecurity.

**Conclusions:**

The mandatory isolation imposed on older adults with subsistence jobs had an impact on their lives and health, increasing their socio-environmental and labor vulnerability.

## Abstract (pt)

**Introducción:**

La inseguridad alimentaria en adultos mayores con empleos de subsistencia ha sido poco explorada para el contexto de pandemia.

**Objetivos:**

Identificar las condiciones de aislamiento por COVID-19, hábitos alimentarios y sintomatología emocional asociados con inseguridad alimentaria en adultos mayores con empleos de subsistencia en Medellín, Colombia, 2021.

**Métodos:**

Estudio transversal con fuentes primarias de información de 208 trabajadores. El diseño y la ejecución del trabajo de campo se realizaron en colaboración con los trabajadores y sus líderes. Se presentan resultados del componente nutricional de los trabajadores con ≥ 60 años, participantes en el macroproyecto del cual se deriva este producto.

**Resultados:**

68,7% tenía entre 60 y 69 años, y 73,0% era cabeza de familia. Las mujeres reportaron menores ingresos, 53,0% sin pareja, y 58,6% sin permiso para trabajar. Mayor consumo de cigarrillo y licor entre los hombres. El 97,0% se aisló en cuarentena, 83,1% recibió algún tipo de apoyo en pandemia, y 62,2% continuaba recibiéndolo. El 56% presentaba sobrepeso u obesidad, y 32,7% disfuncionalidad familiar severa. El 50% vio afectada su vida, y 96,6% su labor en el aislamiento obligatorio. El 38,0% presentó inseguridad alimentaria moderada/severa, y ésta fue mayor (p < 0,05) en quienes no tenían pareja, consumían una o dos comidas diariamente y disfuncionalidad familiar. Aportaron a explicar inseguridad alimentaria la disfuncionalidad familiar y consumir una o dos comidas diariamente.

**Conclusiones:**

El aislamiento obligatorio impuesto a adultos mayores con empleos de subsistencia generó afectaciones en su vida y salud, aumentando su vulnerabilidad socioambiental y laboral.

## Introduction

The primary goal of food security is to ensure physical, economic, and social access to safe, harmless, and nutritious food in sufficient and appropriate quantities to meet individuals’ nutritional requirements and food preferences.^1^ However, maintaining food security has been a challenge in Latin America and the Caribbean, where moderate/severe food insecurity (MSFI) increased by 16.0% between 2014 to 2020. More than half of this increase occurred in 2020 alone, in the context of the COVID-19 pandemic, when the prevalence of MSFI reached 41%, which represented a 10% increase compared to 2018 (31.0%) ­­— the highest increase across all world regions.^2^ Furthermore, this situation may have intensified even further among older adults working in subsistence jobs on the streets and sidewalks, becoming a matter of concern not only for public health but also for governments and international organizations, since sufficient and adequate nutrition is an essential aspect of human development.

Household food insecurity has been assessed in the region using the Latin American and Caribbean Food Security Scale (Escala Latinoamericana y Caribeña de Seguridad Alimentaria y Nutricional, ELCSA), in order to identify and classify the type of food and nutritional insecurity present in households.^3^ This tool has been used in Colombia since 2010 through the National Survey of Nutritional Status (Encuesta Nacional de Situación Nutricional),^4^ which found a food insecurity rate of 57.7% that year among the population aged 18 to 64, while in 2015 this rate was 54.2% for the same age group.

According to data from the Institute of Urban Studies of Universidad Nacional de Colombia, the use of ELCSA in 11 capital cities revealed that, between 2021 and 2022, 71% of Colombian households experienced food insecurity, with 26% classified as moderate and 16% as severe.^5^ These figures are similar to those reported in the 2022 State of Food Security and Nutrition in the World report, which showed that, in 2021, Latin America and the Caribbean had a prevalence of 26.4% for moderate food insecurity and 14.2% for severe food insecurity.^6^

Family income plays a key role among the various factors that threaten household food insecurity.^6^ Poverty is a particularly concerning issue: according to the National Administrative Department of Statistics (Departamento Administrativo Nacional de Estadísticas), in 2021, 39.3% of Colombian households were living in monetary poverty, and 12.2% in extreme poverty.^7^,^8^ This issue is closely related to informal employment, which serves as a means of survival to cope with lack of opportunities for individuals facing difficult living and working conditions,^9^,^10^ and reached a rate of 58.1% in 2022.^10^

Even though surveys and studies on food and nutritional insecurity in Colombia and other Latin American countries^4^,^5^,^10^-^14^ indicate that it mostly affects the poorest individuals^15^-^17^ — with some even experiencing malnutrition^14^ and mistreatment^18^ —in-depth studies focusing on the eating habits and food insecurity among subsistence workers remain scarce. Research is even rarer when it comes to older adults who must work all day just to afford dinner and who may have been further impacted by the mandatory isolation and lockdown measures imposed during the pandemic, which exposed their socio-environmental and occupational vulnerability as they were forced to interrupt their daily activities due to COVID-19.

For the reasons outlined above, this study aimed to identify the sociodemographic, occupational, and nutritional conditions associated with food insecurity in older adults (≥ 60 years) with subsistence jobs, specifically street vendors, in the city of Medellín during the pandemic in 2021. The main purpose was to generate evidence to support the planning of actions that positively impact older adults working in subsistence activities both in Medellín and in other cities across the country during exceptional circumstances and, more broadly, to contribute to the improvement of their living and health conditions.

## Methods

### Design

This was a cross-sectional study using primary data sources that stems from the main project “Living, working, and health conditions among informal street vendors in Medellín during and after the pandemic (2021-2022),” approved by the Institutional Human Ethics Committee of Universidad CES, Medellín under record no.156, code 965, of 2021. This paper shows findings related to nutritional and dietary aspects and to COVID-19 related isolation conditions among the 208 older informal workers (≥ 60 years) who participated in the main study. This study was classified as posing minimal risk according to Resolution No. 8,430 and was conducted in line with international standards for research involving vulnerable populations, as established by the Council for International Organizations of Medical Sciences.

Through snowball sampling, 656 informal street vendors from the city of Medellín and the district (*corregimiento*) of San Antonio de Prado. The workers were invited by their leaders and by the primary investigator at their market stall, as well as during union meetings and assemblies. An interviewer-assisted survey was administered at a union headquarter and at other community and social venues across the city, including the *corregimiento* (district) of San Antonio de Prado, after its form and content was validated with a group of work leaders and participants.

### INCLUSION CRITERIA

The study included workers aged ≥ 60 years with ≥ 3 years of professional experience, who were informed about the study, its procedures, benefits, scopes, limitations, and who voluntarily agreed to participate after reading and understanding the informed consent prior to data collection. No participant was excluded based on these criteria.

### VARIABLES

#### Dependent variable

MSFI was assessed using the ELCSA,^3^ a 15-item scale with dichotomous (yes/no) responses designed to screen households at risk for food insecurity in accordance with the recommendations of the Food and Agriculture Organization of the United Nations. In the context of this study, since 73% of participants were the primary income earners in their households, nearly 90% were in a position to provide the most accurate account of their household situation. ELCSA scores were categorized as follows: 0: food security; from 1 to 5: mild food insecurity; from 6 to 10: moderate food insecurity; and from 11 to 15 points: severe food insecurity. For bivariate and multivariate analyses, food insecurity was recategorized into two groups: 1) MSFI and 2) food security or mild food insecurity.

#### Independent variables

The study evaluated the following sociodemographic and economic variables: sex; age (60-69/≥ 70 years); monthly income in Colombian pesos (COP); number of dependents; educational level in years; primary income earner in the household (yes/no); marital status (without a partner/with a partner); and work permit status (yes/no).

### Isolation, isolation conditions, and support received during the pandemic

The following variables related to isolation were recorded: whether the person self-isolated; duration of isolation; support received during and after mandatory isolation; type of support received; sources of support; duration and frequency of support; how mandatory isolation after workers life and work.

### Lifestyle, dietary habits, body mass index, and emotional symptoms

Data were collected about number of meals per day, smoking (yes/no), alcohol consumption (yes/no), appetite (poor, fair, good), and BMI status (underweight, normal weight, overweight, and obesity).

### Emotional symptoms

The analysis of emotional symptoms included: impact of emotional state on eating habits; family dysfunction, assessed using the Family APGAR tool and reclassified into: 1) severe and 2) absent/mild/moderate; and depressive symptoms, evaluated using the Zung Depression Scale and reclassified into: 1) moderate/severe and 2) absent/mild.

### Bias control

A total of 208 older adult workers from the main study were included using a census-based approach. The data collection tool was validated for content and format by experts on the subject, workers, and their leaders. Researchers and the interviewer received standardized training, and both the tool and data collection procedures were adjusted based on a pilot test with eight workers.

### Analysis

A descriptive analysis was performed using absolute frequencies and percentages for the dependent variable and for all independent variables. Descriptive statistics — mean, standard deviation, median (Med), interquartile range (IQR), CI for means, and normality tests — for the variables age, average monthly income, educational level, and number of dependents. Bivariate analyses included chi-square association tests and calculation of prevalence ratios (PR) and their respective 95%CI to assess the association between MSFI and independent variables. Multivariate analysis was performed using binomial regression to identify the factors contributing to MSFI in workers’ households. Explanatory variables were included based on the Hosmer–Lemeshow criterion (p < 0.25). All statistical tests were conducted considering a 95% confidence level and a 5% margin of error. Calculations were performed using SPSS®, version 26 (license by the Universidad de Antioquia), and Epidat 3.1.

## Results

### Sociodemographic and economic conditions

The majority of the study population (68.7%) was male, with a male-to-female ratio of 2.2:1, and 72.6% were between 60 and 69 years old (Med = 65, IQR = 7). Moreover, 73% were the primary income earner, with a higher percentage among women (75.4%). The average monthly income was higher among men. Only 57.2% reported having a partner and, by sex, 80% of women did not have a partner (Tables 1 and 2).

**Table 1:** Sociodemographic, economic, working, and isolation conditions, and support received by informal street vendors during isolation according to workers’ sex, Medellín, 2020-2021

Condition/characteristic	Sex				Total	
	Male		Female			
	n	%	n	%	n	%
Age (years)						
60-69	101	70.6	50	76.9	151	72.6
≥ 70	42	29.4	15	23.1	57	27.4
Primary income earner						
Yes	103	72.5	49	75.4	152	73.0
No	39	27.3	16	24.6	55	27.0
Marital status						
Without a partner	67	47.0	52	80.0	119	57.2
With a partner	76	53.0	13	20.0	89	42.8
Work permit status						
Yes	87	60.8	35	53.8	122	58.6
No	56	39.2	30	46.2	86	41.4
Self-isolated during mandatory quarantine						
Yes	137	96.0	65	100.0	202	97.0
No	6	4.0	0	0.0	6	3.0
Duration of mandatory isolation (weeks)						
≤ 4	12	8.7	3	4.6	15	7.4
5-12	20	15.0	9	13.8	29	14.3
> 12	105	77.8	53	81.5	158	78.2
Received support during mandatory quarantine						
Yes	115	81.0	57	88.0	172	83.1
No	27	19.0	8	12.0	35	16.9
Types of support received during mandatory quarantine						
Monetary aid						
Yes	49	42.2	25	43.1	74	42.5
No	67	57.8	33	56.9	100	57.5
Complete market						
Yes	65	56.0	35	60.4	100	57.5
No	51	44.0	23	39.6	74	42.5
Food support						
Yes	29	25.0	15	25.8	44	25.3
No	87	75.0	43	74.2	130	74.7
Rental Payment Support						
Yes	17	14.6	5	8.6	22	12.6
No	99	85.4	53	91.4	152	87.4
How mandatory isolation affected participants’ lives						
Completely	12	8.4	16	24.6	28	12.5
Greatly	50	35.0	28	43.1	78	37.5
Moderately	24	16.8	8	12.3	32	15.4
Slightly	24	16.8	7	10.7	31	15.0
Not at all	33	23.0	6	9.2	39	18.8
How mandatory isolation affected participants’ work						
Completely	124	86.7	63	96.9	187	89.9
Greatly	12	8.4	2	3.1	14	6.7
Slightly or nothing at all	7	4.9	0	0.0	7	3.4
Reported needing food aid program						
Yes	133	93.0	59	90.7	192	92.3
No	10	7.0	6	9.3	16	7.7
Received food aid after mandatory isolation						
Yes	88	61.5	47	73.4	135	62.2
No	55	38.5	17	26.6	72	34.8
Who provided the support						
Social Welfare Department	11	12.8	5	10.4	16	11.9
Program. AAM-MC	2	2.3	1	2.1	3	2.2
Private institution	1	1.2	5	10.4	6	4.5
University	71	82.5	36	75.0	107	79.9
Other	1	1.2	1	2.1	2	1.5
Frequency of support						
Once a week	1	25.0	0	0.0	1	11.1
Every 2 or 3 weeks	2	50.0	3	60.0	5	55.5
Once a month	1	25.0	2	40.0	3	33.3
Duration of support						
1 day	22	19.0	8	14.0	30	17.3
1-4 weeks	14	12.0	3	5.3	17	9.8
2-5 months	75	64.6	44	77.2	119	68.8
> 6 months	5	4.3	2	3.5	7	4.0

AAM-MC = older adult assistance program for female heads of household (*atención al adulto mayor, mujer cabeza de familia*).

**Table 2:** Descriptive statistics of sociodemographic conditions of older adults with subsistence jobs participating in the study, according to sex (n = 208), Medellín, 2020-2021

Variable	Mean (SD)	Med (IQR)	95%CI	K-S normality test
Sociodemographic conditions of overall workers				
Age (years)	66.8 (5.63)	65 (8)	66.0-67.5	0.008
Monthly income (COP)	482,692 (282,738)	400,000 (420,000)	444,042-521,342	0.000
Educational level (years)	3.9 (3.1)	3 (3)	3.5-4.3	0.000
Number of dependents	2.0 (1.9)	1.0 (2.0)	1.7-2.2	0.000
Sociodemographic conditions of male workers				
Age (years)	67.1 (5.8)	65 (7)	66.1-68.0	0.150
Monthly income (COP)	534,195 (293,388)	500,000 (400,000)	485,696-582,695	0.000
Educational level (years)	4 (3)	4 (3)	3.5-4.5	0.000
Number of dependents	2 (1.9)	1 (2)	1.64-2.27	0.000
Sociodemographic conditions of female workers				
Age (years)	66.1 (5.2)	65 (7)	64.8-67.4	0.200
Monthly income (COP)	369,384 (220,474)	300,000 (350,000)	314,753-424,015	0.000
Educational level (years)	3.65 (3.33)	3 (4)	2.82-4.47	0.000
Number of dependents	2 (1.8)	2 (3)	1.52-2.41	0.000

95%CI = 95% confidence interval; COP = Colombian pesos; IQR = interquartile range; K-S = Kolmogorov–Smirnov; Med = median; SD = standard deviation. *The data are normally distributed if p > 0.05.

Half of the workers had 3 years of education or less, with men exhibiting higher levels of education. On average, workers had two dependents, and women had more dependents than men (Me = 2) (Table 2).

### Isolation conditions and support received during the pandemic

A total of 97% self-isolated during mandatory quarantine. Of these, more than 78% remained in isolation for more than 12 weeks, while 14.3% did so between 5 and 12 weeks. The main forms of support received included monetary aid (42.5%), groceries or food supplies (57.5%), or benefits from the state’s elderly care program (12.6%), with similar rates among men and women (Table 1).

Half of the study participants stated that their lives were affected by the mandatory isolation: 12.5% completely and 37.5% greatly, with women being the most affected. Concerning their work, 89.9% reported it was completely affected, and 6.7% greatly affected, with all women reporting being affected during this period (Table 1).

Furthermore, 92.3% said they needed the food aid program, and 62.2% continued receiving such support after mandatory isolation, predominantly women (73.4%). Additionally, 79.9% reported receiving support from a private university in the city, and 11.9% from the Social Welfare Department. Most of this support was delivered every 2 or 3 weeks (55.5%) or once a month (33.3%). Finally, 68.8% of participants received support for 2 to 5 months, whereas 17.3% reported having received it for only 1 day (Table 1).

### Eating habits, food insecurity, body mass index, appetite, and emotional symptoms

Overall, 30.8% of participants had two meals a day, and 58.6% had three, mostly men. A greater proportion of women (9.2%) had only one meal a day. Moreover, 72.1% reported having a good appetite; however, this was less frequent for women, among whom 30.8% described their appetite as fair and 10.7% as poor. Overweight was observed in 38.6%, and obesity in 17.4% of workers, with the latter being more prevalent among women (28.1%). Finally, 19.3% reported alcohol consumption and 25% smoking, both habits being more common among men (Table 3).

**Table 3:** Workers’ eating habits, food insecurity, BMI, and emotional symptoms according to sex, Medellín, 2020-2021

Variable	Sex				Total	
	Men		Women			
	n	%	n	%	n	%
Number of meals per day						
1	5	3.5	6	9.2	11	5.3
2	44	30.8	20	30.8	64	30.8
3	89	62.2	33	50.8	122	58.6
> 3	5	3.5	6	9.2	11	5.3
Alcohol consumption						
Yes	36	25.4	4	6.1	40	19.3
No	106	74.6	61	93.9	167	80.7
Smoking						
Yes	41	28.7	11	16.9	52	25.0
No	102	71.3	54	83.1	156	75.0
Appetite						
Good	112	78.3	38	58.5	150	72.1
Fair	29	20.3	20	30.8	49	23.5
Poor	2	1.4	7	10.7	9	4.4
BMI status						
Low weight	6	4.2	1	1.6	7	3.4
Normal weight	63	44.0	21	32.8	84	40.6
Overweight	56	39.2	24	37.5	80	38.6
Obesity	18	12.6	18	28.1	36	17.4
Food insecurity according to the ELCSA scale						
Food security	34	57.6	16	53.4	50	48.5
Mild food insecurity	6	10.2	4	13.3	10	9.7
Moderate food insecurity	8	13.6	4	13.3	12	11.7
Severe food insecurity	11	18.6	6	20.0	31	30.1
Food insecurity according to the ELCSA scale (two categories)						
Moderate/severe food insecurity	52	36.4	27	41.5	79	38.0
Mild food insecurity/food security	91	63.6	38	58.5	129	62.0
Family dysfunction						
Severe	46	32.2	22	33.8	68	32.7
Absent/mild/moderate	97	67.8	43	66.2	140	67.3
Depressive symptoms						
Moderate/severe	3	2.1	0	0.0	3	1.5
Absent/mild	140	97.9	65	100	205	98.5
Emotional state affects food consumption						
Yes	28	19.6	27	41.5	55	26.5
No	115	80.4	38	58.5	153	73.5

BMI = body mass index; ELCSA = Escala Latinoamericana y Caribeña de Seguridad Alimentaria y Nutricional.

### Emotional symptoms

A total of 26.5% of study participants reported that their emotional state affected food consumption, a pattern that was more pronounced among women (41.5%). In relation to depressive symptoms, they were moderate or severe in only 1.5% of workers, whereas 32.7% showed severe family dysfunction (Table 3).

### Food insecurity

Concerning household food insecurity, 30.1% of workers experienced severe levels, and 11.7% experienced moderate levels, based on the ELCSA scale, with similar percentages among men and women. When recategorized, the scale revealed that 38.8% of them presented MSFI (Table 3).

### Sociodemographic and mandatory isolation conditions associated with household food insecurity among workers

A statistically significant association (p < 0.05) was found between MSFI in this working population and their marital status, with those without a partner showing a 61% higher prevalence (PR = 1.61, 95%CI = 1.10-2.38). Although the other conditions explored were not statistically significant, a higher MSFI prevalence were observed among individuals aged 60-69 years and among women (Table 4).

**Table 4:** Sociodemographic and mandatory isolation conditions associated with food insecurity among older adults participating in the study, Medellín, 2020-2021

Condition/characteristic	MSFI		Total	Chi-square test (p < 0.05)	PR (95%CI)
	Yes n (%)	No n (%)			
Biological sex					
Female	27 (41.5)	38 (58.5)	65	0.50 (0.476)	1.14 (0.80-1.64)
Male	52 (36.4)	91 (63.6)	143		1.0
Recategorized age (years)					
60 a 69	60 (39.7)	91 (60.3)	151	0.71 (0.396)	1.19 (0.79-1.81)
≥ 70	19 (33.3)	38 (66.7)	57		1.0
Marital status					
Without a partner	54 (45.4)	65 (54.6)	119	*6.46 (0.011)	1.61 (1.10-2.38)
With a partner	25 (28)	64 (72)	89		1.0
Primary income earner					
Yes	54 (35.5)	98 (64.5)	152	1.13 (0.287)	0.81 (0.56-1.18)
No	24 (43.63)	31 (56.37)	55		1.0
Mandatory isolation conditions					
Self-isolated during quarantine					
Yes	75 (37.1)	127 (62.9)	202	2.12 (0.141)	0.55 (0.30-1.00)
No	4 (67)	2 (33)	6		1.0
Duration of isolation (weeks)					
≤ 4	3 (40)	12 (60)	15	2.46 (0.482)	1.0
5-8	5 (45.4)	6 (54.6)	11		2.27 (0.68-7.55)
9-12	6 (33.3)	12 (66.7)	18		1.66 (0.50-5.55)
≥ 12	61 (38.6)	97 (61.4)	158		1.93 (0.69-5.41)
Received support during the mandatory isolation					
Yes	65 (37.8)	107 (62.2)	172	0.00 (0.942)	0.98 (0.61-1.57)
No	13 (37.1)	22 (62.9)	35		1.0
Type of support during mandatory isolation: monetary					
Yes	28 (37.8)	46 (62.2)	74	0.00 (0.982)	1.00 (0.68-1.47)
No	38 (38)	62 (62)	100		1.0
Type of support during mandatory isolation: food aid and groceries					
Yes	38 (38)	62 (62)	100	0.00 (0.982)	0.99 (0.67-1.46)
No	28 (37.8)	46 (62.2)	74		1.0
Type of support during mandatory isolation: rental payment agreement					
Yes	12 (54.5)	10 (45.5)	22	2.95 (0.085)	1.53 (0.99-2.38)
No	54 (35.5)	98 (64.5)	152		1.0
Duration of support received during isolation					
< 2 weeks	14 (41.2)	20 (58.8)	34	1.05 (0.901)	1.44 (0.42-4.98)
1 month	6 (46.1)	7 (53.9)	13		1.62 (0.44-5.99)
2-3 months	38 (36.2)	67 (63.8)	107		1.27 (0.38-4.20)
4-5 months	6 (42.8)	8 (57.2)	14		1.50 (0.40-5.60)
≥ 6 months	2 (28.6)	5 (71.4)	7		1.00
Received food assistance from the state during and after mandatory isolation due to the pandemic					
No	30 (41.6)	42 (58.4)	72	0.74 (0.387)	1.17 (0.82-1.67)
Yes	48 (35.5)	87 (64.5)	135		1.00

MSFI = moderate/severe food insecurity; PR = prevalence ratio. *Significant statistical association when p < 0.05.

There was no statistically significant association between mandatory isolation conditions and prevalence of MSFI. Nevertheless, higher prevalences were observed for duration of isolation, type of support received, and duration of support. Specifically, the prevalence of MSFI was 1.27 times higher among workers isolated for 5 to 8 weeks (PR = 2.27), 66% higher among those isolated for 9 to 12 weeks (PR = 1.66), and 93% higher among those isolated for more than 12 weeks (PR = 1.93) (Table 4).

Furthermore, the prevalence of this condition was 53% higher among workers who needed to negotiate a rental payment agreement for their residence, of those who received support during quarantine, 62.2% received support for less than 2 weeks, 1 month, 2 to 3 months, and 4 to 5 months (Table 4).

### Association of eating habits, emotional symptoms, and body mass index with household food insecurity among workers

Significant associations (p < 0.05) were observed between the prevalence of MSFI and the number of meals a day and family dysfunction. Individuals who had one or two meals a day showed a 91% higher prevalence compared with those who had more than two meals a day (PR = 1.91; 95%CI = 1.36-2.68). MSFI was also 63% more prevalent in workers experiencing severe family dysfunction (PR = 1.63; 95%CI = 1.17-2.19) (Table 5).

**Table 5:** Association of food insecurity with eating habits, BMI, and family dysfunction in older adults participating in the study, Medellín, 2020-2021

Condition/characteristic	MSFI		Total	Chi-square test (p < 0.05)*	PR (95%CI)
	Yes n (%)	No n (%)			
BMI					
Low weight	1 (14.3)	6 (85.7)	7	2.66 (0.447)	0.32 (0.05-2.04)
Normal weight	33 (39.2)	51 (60.8)	84		0.88 (0.56-1.39)
Overweight	28 (35)	52 (65)	80		0.79 (0.49-1.26)
Obesity	16 (44.4)	20 (55.6)	36		1.00
Alcohol consumption					
Yes	12 (30)	28 (70)	40	1.24 (0.264)	0.76 (0.46-1.26)
No	66 (39.5)	101 (60.5)	167		1.00
Smoking					
Yes	24 (46.1)	28 (53.1)	52	1.96 (0.160)	1.30 (0.91-1.88)
No	55 (35.2)	101 (64.8)	156		1.00
Appetite					
Fair	20 (40.8)	29 (59.2)	49	0.27 (0.871)	1.09 (0.74-1.62)
Poor	3 (33.3)	6 (66.7)	9		0.59 (0.35-2.30)
Good	56 (37.3)	94 (62.7)	150		1.0
Number of meals a day					
1-2	41 (51.5)	34 (48.5)	75	13.86 (0.000)	1.91 (1.36-2.68)
≥ 3	38 (30.3)	95 (69.7)	133		1.00
Has regular meal times					
No	47 (39.5)	72 (60.5)	119	0.27 (0.602)	1.10 (0.77-1.57)
Yes	32 (35.9)	57 (64.1)	89		1.0
Received state food aid during the pandemic					
No	30 (41.6)	42 (58.4)	72	0.74 (0.387)	1.17 (0.82-1.67)
Yes	48 (35.5)	87 (64.5)	135		1.0
Source of support during the pandemic					
Welfare Social Department	5 (31.2)	11 (68.8)	16	0.70 (0.951)	0.84 (0.39-1.80)
Prog. AM-MCF	1 (33.3)	2 (66.7)	3		0.89 (0.18-4.50)
Private organization	3 (50)	3 (50)	6		1.34 (0.58-3.09)
Other organizations	1 (33.3)	2 (66.7)	3		0.89 (0.18-4.50)
City university	40 (37.3)	67 (62.7)	107		1.0
Family dysfunction					
Severe	35 (51.4)	33 (48.6)	68	7.80 (0.005)	1.63 (1.17-2.29)
Moderate/mild/absent	44 (31.4)	96 (68.6)	140		1.0

BMI = body mass index; MSFI = moderate/severe food insecurity; PR = prevalence ratio; Prog. AM-MCF = older adult assistance program for female heads of household (*adulto mayor, mujer cabeza de hogar*). * Significant statistical association when p < 0.05.

Although not statistically significant, higher prevalences of MSFI were observed among individuals who received food aid during mandatory isolation from a private organization, among smokers, among those who received state food aid during the pandemic, and among those who have regular meal times (Table 5).

### Conditions that contributed to explaining household MSFI among workers

When adjusting MSFI for the variables that showed a statistically significant association in bivariate analysis or p-values < 0.25, in order to determine which of them collectively explained a higher or lower prevalence of MSFI, it was found that moderate/severe family dysfunction significantly explained an 1.07 times higher prevalence of MSFI (adjusted PR = 2.07, 95%CI = 1.17-3.67) and having one or two meals a day explained a 65% higher prevalence of MSFI (adjusted PR = 1.65, 95%CI = 0.99-2.77) when adjusted by marital status, smoking, self-isolation during quarantine, and needing to negotiate a rental payment agreement for their residence (Table 6). Although not statistically significant, the absence of a partner and smoking still contributed to a higher prevalence of food insecurity (Table 6).

**Table 6:** Lifestyle and eating habits that contribute to explain food insecurity in older adults participating in the study, Medellín, 2020-2021

Condition/characteristic	95%CI			95%CI		
	Crude PR	LT	UT	Adjusted PR	LT	UT
Marital status	1.61	1.10	2.38	1.24	0.73	2.09
Smoking – Yes	1.30	0.91	1.88	1.37	0.80	2.34
Number of meals a day – One or two	1.91	1.36	2.68	1.65	0.99	2.77
Self-isolated during quarantine – Yes	0.55	0.30	1.00	0.40	0.08	2.07
Support during mandatory isolation						
Rental payment agreement – Yes	1.53	0.99	2.38	1.00	N.C	N.C
Family dysfunction – Moderate/severe	1.63	2.17	2.29	2.07	1.17	3.67

CI = confidence interval; LT = lower threshold; N.C. = Not calculated ; PR = prevalence ratio.

## Discussion

In this study, the working population of older adults with subsistence jobs during the pandemic exhibited sociodemographic and economic conditions that reflect a significant shift, affecting not only the rate of female-headed households but also increasing the loneliness experienced by those who were socially and economically disadvantaged, both during and after the mandatory lockdown. Women had to assume greater caregiving responsibilities but earned a lower income. Although their eating habits and lifestyle could suggest greater vulnerability, the prevalence of MSFI was lower than that observed in the general population and in other working populations assessed before and during the pandemic. This finding may, in part, be explained (at least in this population) by the support received during the pandemic, mainly from private organizations.

This study only included older adults with subsistence jobs on the streets and sidewalks of Medellín and in one of its *corregimientos*; therefore, the results should be interpreted in the context of the study population and in other older adults with similar occupational conditions. Even though older adults may be involved in many informal jobs, the study findings discussed below do not allow for inferences or analyses about other employment scenarios and contexts. Due to the sampling method employed, statistical inferences are limited, and, although these conditions may occur within the study population, the inference error cannot be calculated. Therefore, the results must be interpreted in light of the context and setting where the study was conducted, along with the limitations imposed by the mandatory lockdown period during the pandemic, which further complicated access to this working population.

Although the ELCSA scale is designed to screen for household food insecurity, due to the high level of responsibility and the head-of-household role assumed by these older adults, their responses were deemed to accurately represent the household’s food and nutritional security status in line with this scale, as outlined in the methods.

### Sociodemographic, economic, working, and isolation characteristics, and support received

The study population consisted mainly of men (68.7%) and younger older adults, with 72.6% aged between 60 and 69 years; women were on average younger and also the primary income earners in their household (75.4%). These findings are consistent with those of the 2016 study by Garzón et al.,^19^ in which 77% of the surveyed older street venders were aged 60 to 69 years, with a predominance of men (79%). In turn, a 2020 study by Zuleta^20^ found that 30% (n = 27) of street vendors were aged 18 to 29 years old, and women accounted for 85.6%^20^ of the total. The figures contrast with those reported by Quilaguy & Chaves^21^ in 2020 in a pre-pandemic scenario, where the proportion of male workers of all ages was 52.3%.

It is particularly concerning that the older adults participating in the present study remained the primary income earners in their household and that their average monthly income was below the legal monthly minimum wage in Colombia for 2021, with women earning less than men. Women also had more dependents, which contrasts with findings from Quilaguy & Chaves^21^ showing a lower percentage (67.9%) of workers who were the primary income earners in their household for the general population included.

Additionally, Cano et al.^18^ reported that 54.8% of older adults in Antioquia, Colombia, earned from 566,000 to 575,000 COP, while, before the pandemic, Quilaguy & Chaves^21^ reported incomes from 500,001 to 1,000,000 COP for 40.4% of respondents in Bogotá. These incomes were considerably higher than those reported by the older adults included in this study, with women experiencing an even more critical situation. The workers in this study had at most one dependent, in contrast to the study by Quilaguy & Chaves,^21^ who observed that 67.9% of participants reported having between one and three dependents; however, their study included workers from all age groups. In the present study, half of the women had ≤ 2 dependents, consistent with the findings reported in 2019 by Herrera,^22^ where each street vendor was responsible for an average of 2.36 dependents. Half of the workers participating in the present study had 3 years of education or less, a situation markedly different from that reported by Robledo et al.^23^ where 55.8% of vendors had completed high school.

A notable finding is the high level of loneliness among these older adults, considering that 57.2% had no partner at the time of the survey — which was even more significant in women, of which 80% of whom had not partner —, 97% stated that they had self-isolated during the mandatory quarantine, and 78.2% remained isolated for more than 12 weeks. However, there is currently little literature reporting such data, making it difficult to compare the loneliness experienced by these workers with that of other older adults with subsistence jobs during the pandemic.

For this type of working population, one of the most encouraging forms of support is having a work permit. Nevertheless, 41.4% of them did not have such permit, with an even higher proportion among women (46.2%). This reality contrasts with findings by Miranda et al.^24^ in Quito, Ecuador, who reported that 70% (n = 6,724) of informal vendors during the COVID-19 pandemic had a work permit and were operating under regulated conditions.

Regarding the support received during the pandemic, 83.1% received it during mandatory isolation, and more than 60% continued to receive it, mainly in the form of food or monetary aid. This support was mainly provided by a private university in the city — a more favorable situation than that reported by Ramírez & Aragón^25^ in their study conducted in Cali, Colombia, where only 21% of the surveyed population received support from the government.

In the present study, 37.5% of workers stated that mandatory lockdown greatly affected their lives, and for 89.9% it completely affected work. These figures aligned with those presented by Quilaguy & Chaves^21^ concerning, in which 88.3% of participants reported a decline in quality of life and 79% reported an impact on their professional life. Moreover, Becerra et al.^26^ found that 66% of the respondents in their study experienced a decline in income.

### Eating habits, food insecurity, emotional symptoms, and body mass index

Approximately 60% of the population assessed in the present study stated that they had three meals a day, a behavior more prevalent in men (62.2%), and higher that that observed in a pre-pandemic study of this working population, in which Garzón et al.^15^ showed that 48.8% of street vendors had three meals a day, whereas 44.1% consume one or two meals a day.

One in four workers in the present study smoked, a rate lower than the 31.1% reported by Mendinueta et al.^27^ for the same habit. Furthermore, 20% of workers consumed alcohol; a markedly different scenario described by Mendinueta et al.,^27^ who noted that 81.4% of vendors consumed alcohol. According to BMI classification, 38.6% of our study population was overweight, and 17.4% was obese, findings that differ from the figures observed in two studies by Garzón et al.^19^ in the pre-pandemic period, which showed that 62.2% of older adults^19^ and 68.5%^15^ of the 686 workers included in the studies were overweight or obese. Food security was present in 48.5% of households assessed in our study, a finding in contrast with that of Miranda et al.^24^ in Ecuador, who found that 77.3% of the surveyed population experienced mild or moderate food insecurity.

The results of the present study also showed that 1.5% presented moderate/severe depressive symptoms, which represents a noteworthy finding, since global alerts were issued during the pandemic about the increase in anxiety and depressive symptoms. In this regard, Miranda et al.,^24^ in their study conducted in Ecuador, reported that 19.1% of the informal workers surveyed exhibited moderate/severe depressive symptoms.

Finally, it was observed that food insecurity was significantly higher (p < 0.05) in individuals without a partner, who had one or two meals a day, and who experienced family dysfunction, whereas a higher prevalence of this insecurity can be explained by family dysfunction and having one or two meals a day. However, this evidence is difficult to compare, since existing studies to date have not reported this type of analysis, particularly in older adults with street-based subsistence jobs during the pandemic.

## Conclusions

Mandatory isolation may have had a greater impact on the lives and health of older adults working in subsistence jobs, increasing their socio-environmental and occupational vulnerability. In general, the conditions associated with and contributing to MSFI in the households of these older adult workers can be reversed through public health and occupational health actions implemented by the State in the short, medium, and long term, with the participation and collaboration of workers’ associations, their representatives, and the workers themselves.

This is a topic of public concern for Medellín, Colombia, as well as for other Latin American cities, where accelerated population aging is accompanied by the need for older adults to keep working in subsistence jobs —here, as informal workers on the streets and sidewalks, turning these public spaces into their workplace. This situation also places them at risk of MSFI, endangering not only their own survival but also that of their dependents.
